# Highly stretchable transparent bar coated Ag NW/PEDOT:PSS hybrid electrode for wearable and stretchable devices

**DOI:** 10.1039/d1ra08173j

**Published:** 2022-01-24

**Authors:** Ju-Hyeon Lee, Vivekanandan Raman, Chaeyoung Kang, Hyun-Uk Ha, Hae-Jun Seok, Han-Ki Kim

**Affiliations:** School of Advanced Materials Science and Engineering, Sungkyunkwan University 2066, Seobu-ro, Jangan-gu Suwon-si Gyeonggi-do 16419 Republic of Korea hankikim@skku.edu

## Abstract

In this study, we demonstrated poly(3,4-ethylenedioxythiophene): poly(styrene sulfonate) (PEDOT:PSS) as a composite with Ag nanowire (Ag NW) to enhance the stretchability of the Ag NW network electrode. The composite Ag NW/PEDOT:PSS hybrid ink (AP ink) was prepared at a ratio of 1 : 10, 1 : 20, and 1 : 30, respectively and bar coated on polyurethane substrate. The different ink ratios were studied and optimized with a sheet resistance of 14.93 Ω sq^−1^. and a transmittance of 88.6% showing a high performance in mechanical stress tests such as bending, folding, rolling, twisting, and stretching. It also showed a conductive bridge effect where the PEDOT:PSS acted as an anchor or support to Ag NW during mechanical strain and PEDOT:PSS also enhanced the electrical conductivity of the Ag NW. Therefore, to prove the real time performance of the electrode as a wearable device, we fabricated transparent electroluminescence devices and thin film heater devices which are highly flexible and demonstrated excellent performance proving that the AP electrode is more suitable candidate for future wearable transparent devices.

## Abstract 2

PEDOT:PSS, the highly stretchable and conductive polymer, can enhances the stretchability of Ag NW electrodes when mixed with Ag NW. Because PEDOT:PSS is still connected to the disconnected Ag NWs, the electrode has enhanced stretchability.

## Graphical abstract

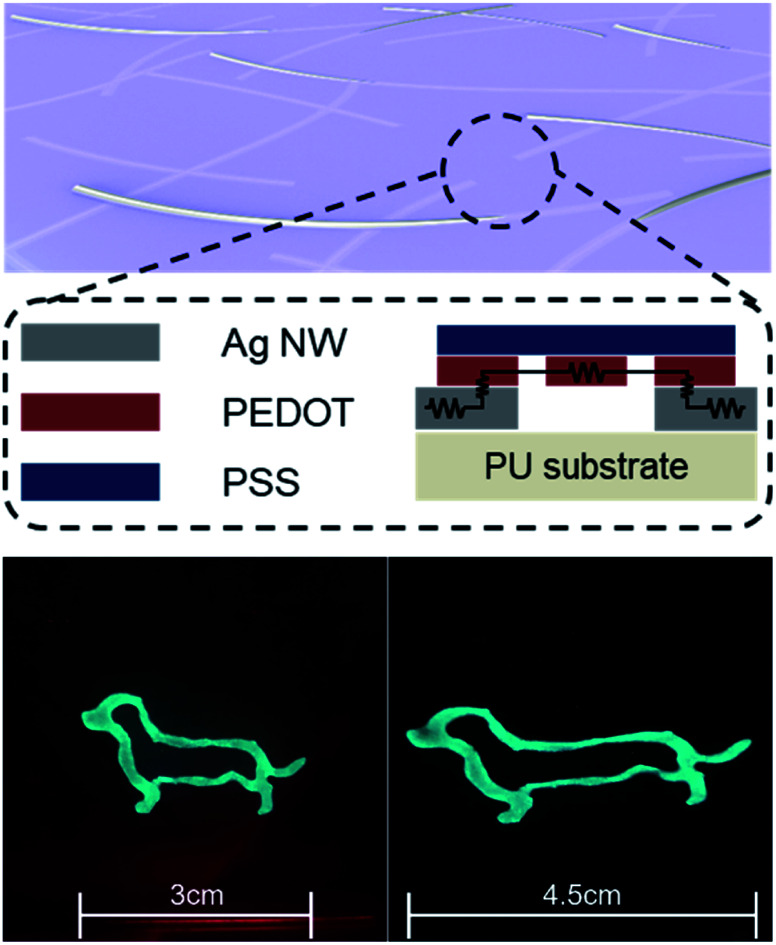

## 1. Introduction

With the advancement of transparent electrode technology, innovations based on wearable portable gadgets have gained popularity for their human convenience and light weight. Therefore, the necessity and urgency for light weight stretchable electrodes is continuously increasing.^1–3^ To fabricate a highly stretchable electrode, the electrode must use surface–substrate manufacturing techniques where the stretchable substrate is coated with the stretchable materials.^4–10^ To date, materials such as silver nanowires (Ag NWs),^11^ carbon nanotubes (CNTs),^12^ graphenes,^13^ and conductive polymers,^14^ poly(3,4-ethylenedioxythiophene):poly(styrene sulfonate) (PEDOT:PSS) have been used for manufacturing flexible, bendable, and stretchable electrodes. Metal nanowires (Ag or Cu) are promising candidates, with Ag NW having the highest electrical conductivity of 6.3 × 10^7^ S m^−1^ among all metal electrodes, including copper (Cu). It is noted that Ag NW has better corrosion resistance than Cu. Moreover, carbon nanotubes (CNTs) have high mechanical stability, flexibility, and transparency though the resistivity is extremely high and also flexible graphene is difficult to manufacture in a large scale with restacking and agglomeration problems.^15^ Although Ag NW has better electrical and optical properties, it has inherent limitations in that the polyvinylpyrrolidone used in Ag NW production introduces an insulating barrier around the Ag NW, increasing the overall sheet resistance of the electrode.^16^ Moreover, common coating strategies of the Ag NW electrode using various solution-based processes such as bar coating,^17^ spray coating,^18^ spin coating^19^ and vacuum filtration techniques^20^ are used to make flexible transparent conducting electrodes (TCEs). For example, Dong *et al.* demonstrated a high performance flexible Ag NW–polyimide (PI) electrode spin coated for non-fullerene solar cell with a power conversion efficiency of 11.6%.^21^ Zhang *et al.* studied Ag NW–PEN (polyethylene naphthalate) spin coated as TCE showed a similar comparable result in transparency and conductivity as commercial indium tin oxide (ITO) electrode.^22^ However, Ag NW has significant disadvantages, including high junction resistance at the contacts of the nanowire, non-uniform morphology, poor mechanical–chemical stability and poor adhesion to the polymer substrate. Meanwhile, although PEDOT:PSS conductive polymers are highly flexible and cheap with a high work function of approximately −5.2 eV, where PEDOT:PSS has been used as a hole conductor, they have a low conductivity and suffer from humidity issues.^23^ However, the majority of the research was focused on conductivity, with flexibility and base conductive change receiving less attention.^24^ Therefore, for studying next-generation stretchable devices, research on how to improve stretchability and increase their resistance to water is needed.

In this study, we developed an Ag NW/PEDOT:PSS composite ink (AP ink) at various mixing ratios and fabricated the electrode *via* bar coating. We suggested a stretchability enhancing mechanism called ‘conductive bridge effect’ by mixing PEDOT:PSS and Ag NW. The Ag NW provided high conductivity while PEDOT:PSS provided high stretchability for the electrode. The network of bare Ag NWs is usually disconnected from stretching. On the other hand, the highly elastic PEDOT:PSS which strongly bonded Ag NWs in AP electrode, interlocked the fractured Ag NWs during heavy stretching without significant interrupting the flow of the current. As a result, the electrode has high electrical property and stretchability. We previously demonstrated that PEDOT:PSS composite with Ag NW can enhance the stretchability of Ag NW electrode with brush painting method.^25^ Though the brush painting method is both simple and cost effective, it is hard to control both the thickness and uniformity control for electrode. Thus, we used bar coating method to control the thickness and uniformity of the electrode by adjusting various parameters such as coating speed, mixing ratio of AP ink, and diameter of coating bar which is called Mayer bar. Moreover, for subjugation of the humidity issue, we added a protective layer called polydimethylsiloxane (PDMS) on top of the AP electrode by spin coating.^23,26^ The PDMS is cost-effectiveness,^27^ high transmittance,^28^ low elastic modulus,^29^ low surface energy,^30^ good insulating property,^31^ and high chemical resistance.^32^ Thus, applying PDMS as a protective layer would seal the electrode as well as reduce the degradation of the electrode. The highly stretchable transparent AP electrode was demonstrated *via* comparing the stretchability of AP electrode and bare Ag NW electrode. The AP electrode endured 10% higher strain and more than twice the repeated stretching test compared to the Ag NW electrode. Moreover, the AP electrode was proved to be highly flexible through 10 000 times of various repetitive mechanical tests.

To verify the applicability of the electrode as wearable devices, the electroluminescence (EL) devices and thin film heater (TFH) devices were manufactured using AP electrode. The EL devices worked well in stretched and after stretching state. The performance of the TFH devices was not degraded in the bent or twisted states, however it was slightly degraded in the stretched state. These results showed the high potential of the cost-effective and easily manufactured AP electrode.

## 2. Experimental

### 2.1. Synthesis and electrode deposition

The hybrid ink was prepared by adding 10 ml of water-based Ag NW solution (average length of 25 μm, average width of 25 nm, Flexio Co., Ltd.) with 1 ml of PEDOT:PSS solution (Clevios PH 1000, Heraeus) and stirring them for 1 hour using a magnetic stirrer. It was then coated using bar coating technique on to a polyurethane (PU) substrate. The samples were then dried at 70 °C for 10 min. The electrode was optimized by changing the hybrid ink ratios of PEDOT:PSS and Ag NW ink to around 1 : 10, 1 : 20, and 1 : 30, respectively. The velocity movement of the bar at 17.5, 35, and 70 mm s^−1^ during bar coating was used to adjust the thickness of the electrode.

### 2.2. Protective layer coating

The fabrication of the hybrid Ag NW/PEDOT:PSS electrode and the protective PDMS (base elastomer: curing agent = 10 : 1) (Sylgard 184, Dow Corning) was mixed into *n*-hexane (Duksan Pharmaceutical) at a ratio of 1 : 2 and the solution was spun coated at 2000 rpm for 30 s on top of the Ag NW/PEDOT:PSS electrode. The PDMS-coated Ag NW/PEDOT:PSS electrode (AP electrode) was then dried in a convection oven at 80 °C for 2 hours.

### 2.3. Material characterization

Electrical and optical properties of AP hybrid electrode were measured using Hall effect measurement (HMS-4500, Ecopia) and UV/vis spectrometer (V-670, Jasco) at a light of wavelength 400 nm to 800 nm, respectively. Surface energy data was collected by measuring the contact angle of droplets of DI water and diiodomethane on samples (Phoenix-MT(A), SEO Co.). The surface morphology of hybrid electrode before and after stretching test were analyzed by field emission-scanning electron microscope (FE-SEM) (JSM-7600F, JEOL). The mechanical flexibility and stretchability of electrodes were measured by laboratory-designed stretching, bending, folding, rolling, and twisting tester. For measuring the stretchability, resistance change of electrodes was noted with increasing strain rate and stretching repetition test at 20% strain. For determining inner/outer critical bending radius of electrodes, resistance change of electrodes was measured by decreasing bending radius until 1 mm. Furthermore, dynamic fatigue tests were used to measure the endurance of AP hybrid electrode. Inner/outer bending fatigue tests were performed 10 000 times at a bending radius of 2 mm. Inner/outer folding tests and rolling tests were done 10 000 times at a bending radius of 3 mm and twisting test was done 10 000 times at a twisting angle of 35°.

### 2.4. Fabrication of stretchable electroluminescence device

For fabricating stretchable EL devices, ZnS:Cu phosphor powder (Shanghai KPT company) and PDMS were mixed at a weight ratio of 2 : 1.^33^ The mixed phosphor ink was brush painted on bottom AP electrode then the top AP electrode was attached and dried at 80 °C for 2 hours in convection oven. The alternating current (AC) potential was introduced to phosphor layer of EL devices using AC power supply.

### 2.5. Fabrication of thin film heater

AP electrode (5 × 2.5 cm) was coated with Ag paste to create contact points on the edges of the samples and dried at 80 °C for 10 min. The Ag contact points were then covered with Cu tape to ensure that they made contact with the power source. The PDMS layer was spin coated to protect the electrode. Direct current (DC) voltage was applied to TFH devices using DC power supply. The temperature was monitored using a thermocouple temperature measuring system that was placed on the surface of the TFH devices. The infrared (IR) thermal images were taken using IR camera tools (A35sc, FLIR).

## 3. Results and discussion

**Fig. 1:**
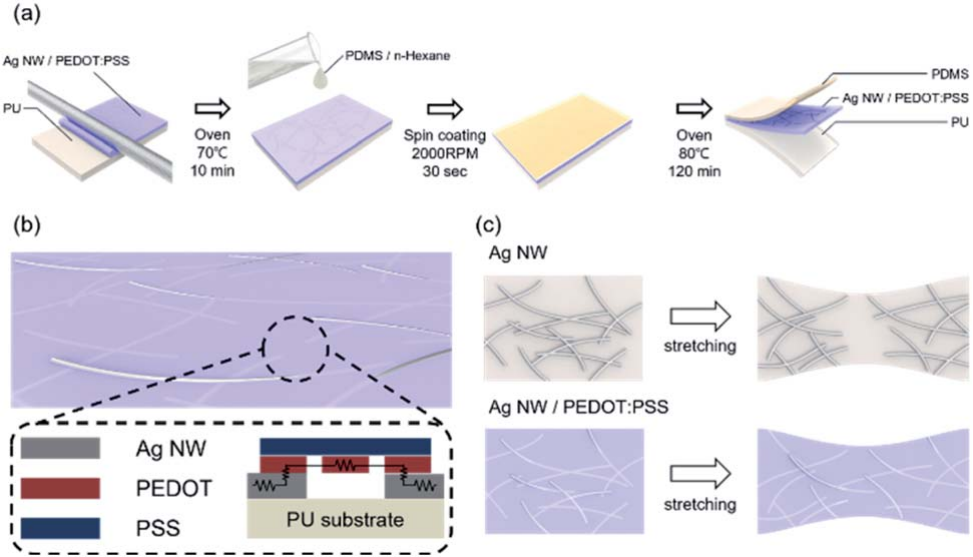
(a) All solution manufacturing process for stretchable PEDOT:PSS/Ag NW hybrid transparent electrode. (b) Schematics of AP electrode structure and conductive bridge effect. (c) Stretchability enhancing mechanism of conductive bridge effect by adding PEDOT:PSS into Ag NW.

Fig. 1a illustrates the bar coated Ag NW/PEDOT:PSS hybrid ink (AP ink) on PU substrate and PDMS coating on top of AP electrode as protective layer. Fig. 1b shows the conductive bridge structure. Because the electrode was connected with both Ag NW and PEDOT:PSS, there were three possible electrical connections, namely Ag NW–Ag NW, Ag NW–PEDOT:PSS, and PEDOT:PSS–PEDOT:PSS. Fig. 1c shows schematics of electrical bridge effect mechanism. Because each Ag NW has a limited ability to stretch itself, an electrode made entirely of Ag NW may be readily disconnected from stretching, as shown in the upper schematic. In contrast, when PEDOT:PSS was incorporated into an Ag NW electrode, the PEDOT:PSS electrically interlinked the Ag NWs even when the electrode was stretched due to its great stretchability. Although the electrical property of PEDOT:PSS was weaker than that of Ag NW, Ag NW served as the primary current channel, and PEDOT:PSS served as an auxiliary electrode for stretchability. As a result, it can be said that Ag NW has a high electrical property and PEDOT:PSS has a high stretchability.

**Fig. 2:**
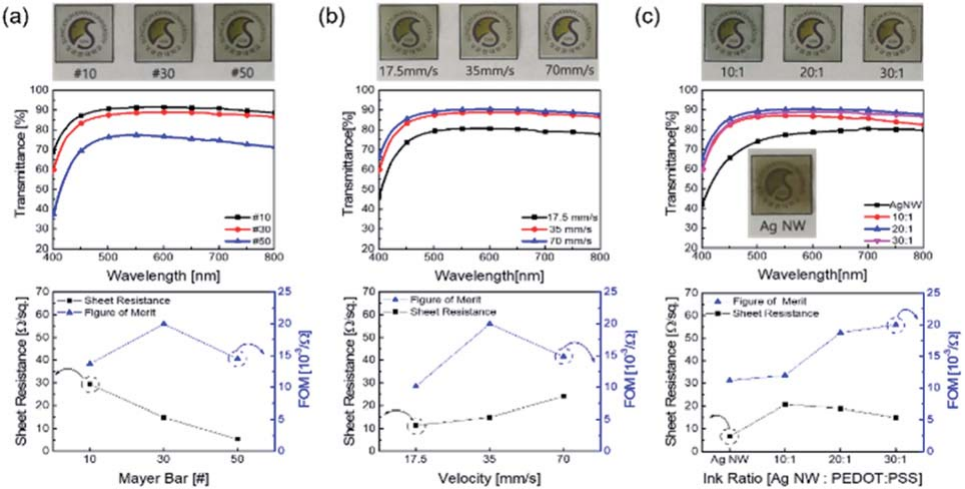
Optical transmittance, sheet resistance and figure of merit as a function of: (a) Mayer bar number, (b) coating velocity, and (c) ink ratio. Top panels showed the optical transparency of each sample.

The Ag NW/PEDOT:PSS ink was tested with various concentration ratios to optimize the electrode. Fig. 2 illustrates the optimization of the Ag NW/PEDOT:PSS electrode by varying the bar winding thickness, coating velocity and the concentration ratios. The optimization was done by comparing the transmittance and the sheet resistance of the variables. To determine the compatible process condition, we used Figure of Merits (FoM) (*T*^10^/*R*_sheet_).^34^ where, *T* is transmittance and *R*_sheet_ is sheet resistance.

Fig. 2a shows the electrode bar coated at different winding thickness and it also shows the transmittance and the sheet resistance of the electrode. As the thickness of the bar winding increases, the transmittance decreases while the sheet resistance decreases. This implied that the coating was non-uniform with greater sheet resistances as the winding thickness decreased, and a winding thickness of 30 # exhibited high transmittance and lower sheet resistance with a larger FoM value. Fig. 2b shows the transmittance and sheet resistance properties of the electrode by varying the coating speed. When the coating speed was reduced, the transmittance decreased. This was due to the slow agglomeration of the solvent as the bar moved slower. But with higher coating velocity, there was an increase in sheet resistance. If the coating velocity was high, less time was needed to coat the ink on the substrate. As a result, the thickness of the electrode was reduced. The thinner film showed an increase in the sheet resistance and transmittance. However, it was observed that there was enough time to coat the ink on the substrate even if the velocity was slower. Thus, it can be said that the slower coating resulted in an abnormally thick electrode. And excessively thick electrode film reduced the sheet resistance and transmittance. In Fig. 2c, the ink ratios of Ag NW and PEDOT:PSS were varied at 10 : 1, 20 : 1, and 30 : 1, respectively. The concentration ratio of 30 : 1 showed excellent potential with an increase in transmittance and a decrease in sheet resistance.

The hydroscopic property of PEDOT:PSS allows humidity to be absorbed in the polymer. Then the PEDOT:PSS expanded, increasing the distance between PEDOTs. As a result, the average hopping distance increased while interactions decreased. The dipole moments of absorbed water can also influence the conductivity of PEDOT:PSS. As such, due to the influence of water, the electrical property of PEDOT:PSS may deteriorate.^23,35^ Also, moisture is the primary cause of silver corrosion. Therefore, water or moisture in the air can have an effect on the reliability of a silver electrode, such as a silver nano wire.^26,36^ In order to protect the electrode from the adverse effects of water, the PDMS was coated on the AP electrode. Because of the Cassie–Baxter state, the PDMS is hydrophobic due to the weak intermolecular interactions known as the London dispersion forces of the methyl group (–CH_3_) in PDMS and the helical molecular structure of PDMS that prohibits methyl groups from being on the same plane.^37–39^

**Fig. 3:**
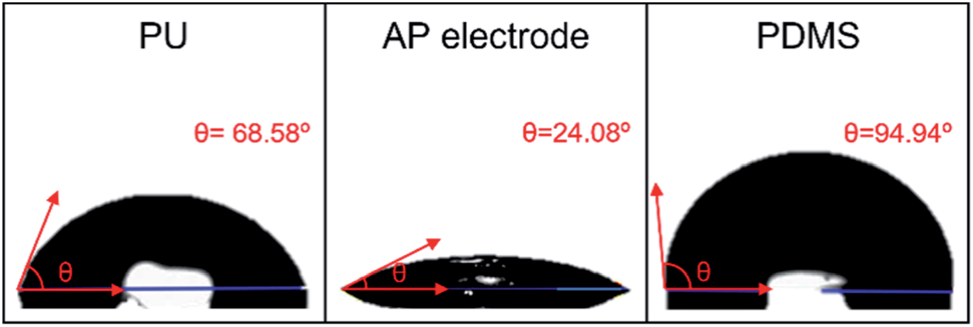
Photography of water droplet on a bare PU substrate, AP electrode on PU substrate, and PDMS on AP electrode.

In addition, Fig. 3 shows the image of DI water droplet on bare PU substrate, optimized AP electrode, and PDMS surface. The contact angle can determine the surface energy.^40–42^ The surface energy of film (*γ*_s_) was calculated with Owens–Wendt method.^43^ The *γ*_s_ can be separated into the dispersion part (*γ*^d^_s_) and the polar part (*γ*^p^_s_) so that each part can be calculated from the contact angle of diiodomethane (DM) for disperse part and deionized water (DI) for polar part using below equations which well-known as Young equation (eqn (1))^44^ and Fowke's method (eqn (2)).^42^

1 *γ*_l_ cos *θ* = *γ*_s_ − *γ*_sl_

2
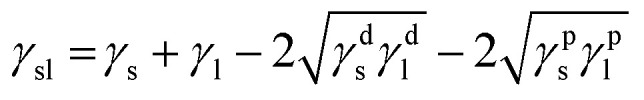


In these equations, *γ* is free energy, under scripts l and s are liquid and solid (film) against their vapor, sl is the interface between liquid and solid, and *θ* is contact angle. The superscripts d and p refer to the dispersion part and the polar part. By plugging eqn (2) into eqn (1), eqn (3) was obtained.3
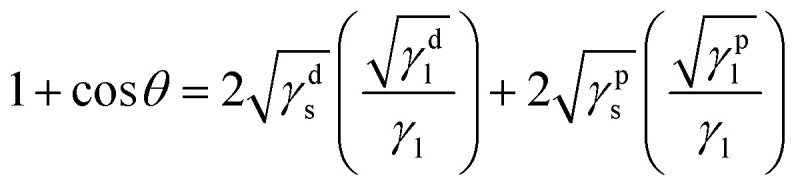


Using the already known *γ*^d^_l_, *γ*^p^_l_, and *γ*_lv_ of DM and DI, the free energy of film can be calculated with contact angles. The results in Table 1 show that the AP electrode had the highest surface energy and PDMS had the lowest surface energy. High surface energy suggested that the AP electrode was highly absorbent and easily washed, limiting the possibility of using the AP electrode for diverse applications such as wearable devices. The hydrophobicity of PDMS allows it to successfully resist against moisture and allows it to be applied to wearable devices.^45^

**Table 1:** Contact angle and surface energy of PU substrate, AP electrode on PU substrate, and PDMS on AP electrode

Surface	Diiodomethane contact angle [°]	Deionized water contact angle [°]	*γ* ^d^_s_ [mJ m^−2^]	*γ* ^p^_s_ [mJ m^−2^]	*γ*_s_ [mJ m^−2^]
PU	30.99	68.58	40.728	7.499	48.227
AP electrode	29.64	24.08	38.054	32.402	70.456
PDMS	50.80	94.94	32.977	0.722	33.699

**Fig. 4:**
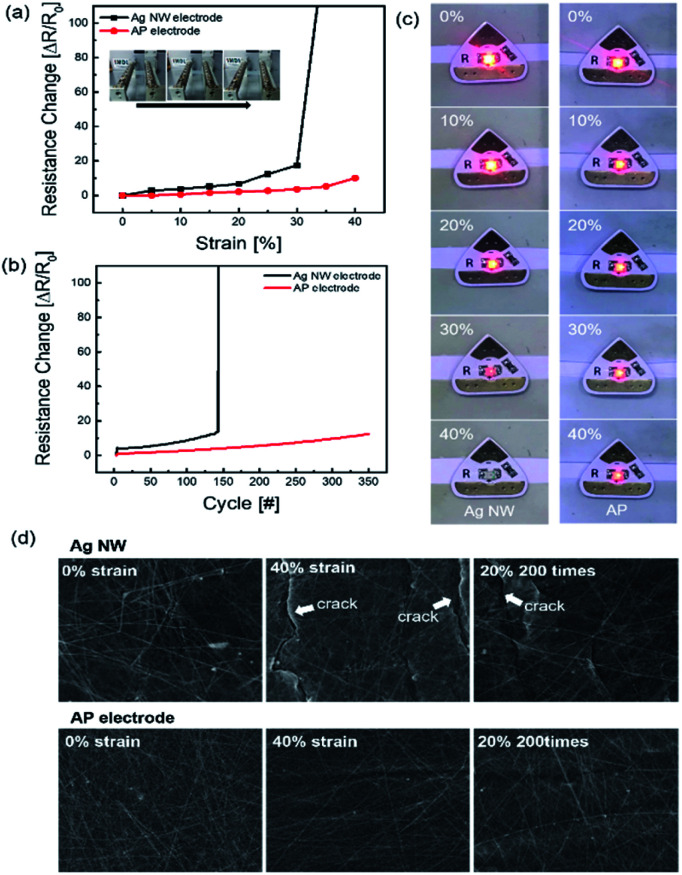
Resistance changes of Ag NW and AP electrodes as a function of: (a) strain and (b) the number of fatigue cycles at 20% strain. (c) Photography of stretching test with LED on bare Ag NW electrode and AP electrode according to the strain with an applied voltage of 5 V. (d) SEM images of Ag NW and AP electrodes after stretching and fatigue test.

The AP electrode was subjected to stretching as illustrated in Fig. 4. To determine the endurance of the stretching electrode, resistance change Δ*R* = (*R* − *R*_0_)/*R*_0_ was calculated where, *R* is the measured resistance and *R*_0_ is the initial resistance. The resistance was tested *in situ* under stretched conditions. Fig. 4a shows the durability of Ag NW electrode and optimized AP electrode for the strain percentage. When the strain rate was increased up to 30%, the Ag NW electrode was broken due to the disconnection of Ag NW network and formed a crack on the electrode film. The AP electrode, on the other hand, was subjected to more than 40% strain, whereas the PEDOT:PSS supported the Ag NW by stretching without breaking the link with the nanowires. As shown in Fig. 4b, AP electrode showed highly enhanced endurance for 350 repetition cycles at a strain of 20%. At 20% strain rate repetition test, Ag NW was broken below 150 cycles. Fig. 4c depicts the enhanced stretching caused by the addition of PEDOT:PSS using a stretching jig and a light emitting diode (LED). When the strain percentage increased, the glowing LED brightness gradually decreased in both Ag NW and AP electrode. However, when stretched to 40%, the Ag NW electrode snapped compared to the AP electrode. The AP electrode was not broken at the higher stretch percentage because PEDOT:PSS helped in securing the bond between individual Ag NWs. The FE-SEM images shown in Fig. 4d clearly denoted the morphology of both the normal electrode and the stretched electrode. The FE-SEM image revealed cracks in the Ag NW electrode at 40% strain, demonstrating that the AP electrode was extremely stretchable, and it lasted at 20% stretch for 200 cycles.

**Fig. 5:**
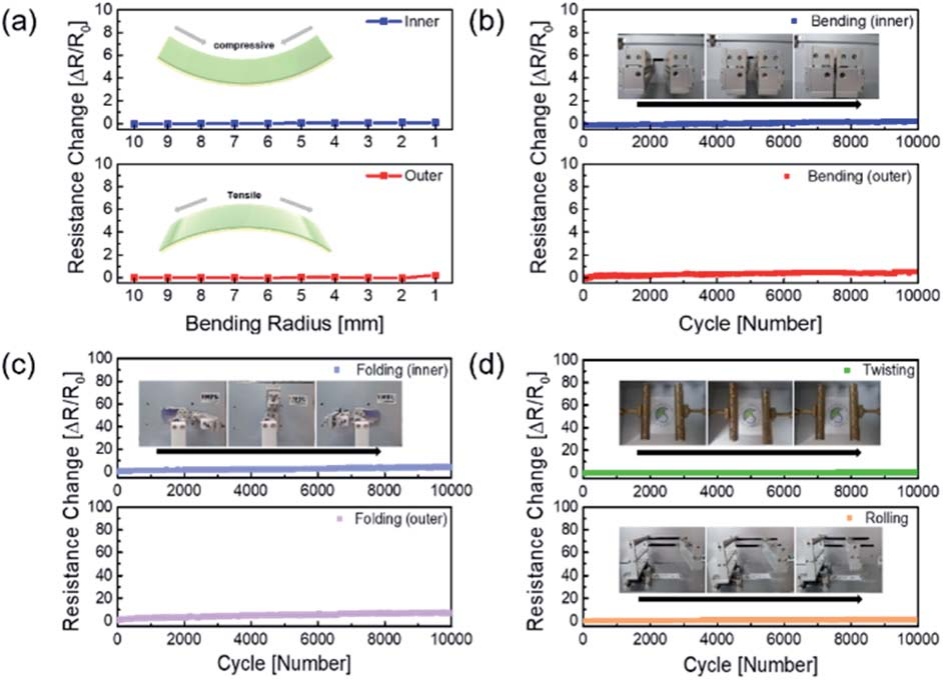
(a) Resistance changes of AP electrode during bending test with decreasing bending radius, (b) inner and outer bending fatigue test for 10 000 times of cycles at a bending radius of 2 mm. (c) Inner and outer folding fatigue test for 10 000 times of cycles at a folding radius of 3 mm. (d) Twisting fatigue test with a twisting angle of 35° and rolling fatigue test at a rolling radius of 3 mm for 10 000 times of cycles, respectively.

The mechanical fatigue test was carried out to further demonstrate the flexibility of the AP electrode in Fig. 5. If the mechanical tests on the Ag NW electrode were repeated, the Ag NW might be brittlely broken as the dislocation moved across the slip plane.^46^ The extremely flexible PEDOT:PSS in the AP electrode, on the other hand, still interlocked the Ag NW despite repeated fatigue tests, and the electrode demonstrated improved mechanical durability. Fig. 5a demonstrates that the outer and inner bending are subjected to tensile and compressive stress, respectively, resulting in no resistance change over the whole bending radius range of 10 mm to 1 mm. Even at 1 mm radius, there was no breaking of the electrodes under tensile and compressive stress, demonstrating that the electrode was exceptionally flexible. Furthermore, the outer and inner bending cycle tests at 2 mm radius for 10 000 cycles revealed minimal resistive change, as illustrated in Fig. 5b. As illustrated in Fig. 5c and d, 10 000 cycles of folding and twisting of the AP electrode resulted in no change in resistance.

**Fig. 6:**
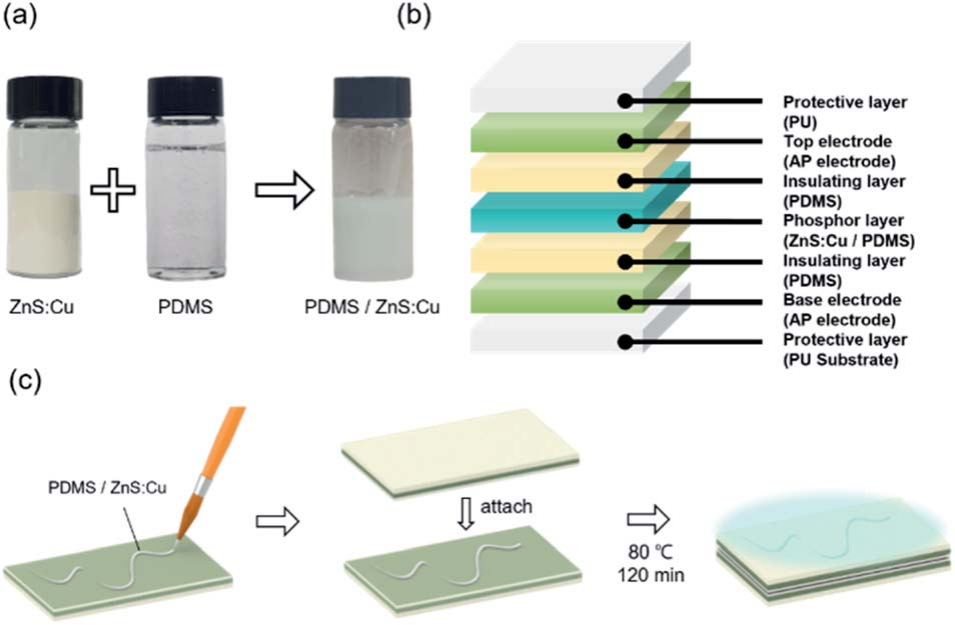
(a) Manufacturing procedures of brush paintable EL ink with ZnS:Cu phosphor powder and PDMS, (b) schematics of inorganic EL device structure, (c) fabrication process of EL device with AP electrode.

**Fig. 7:**
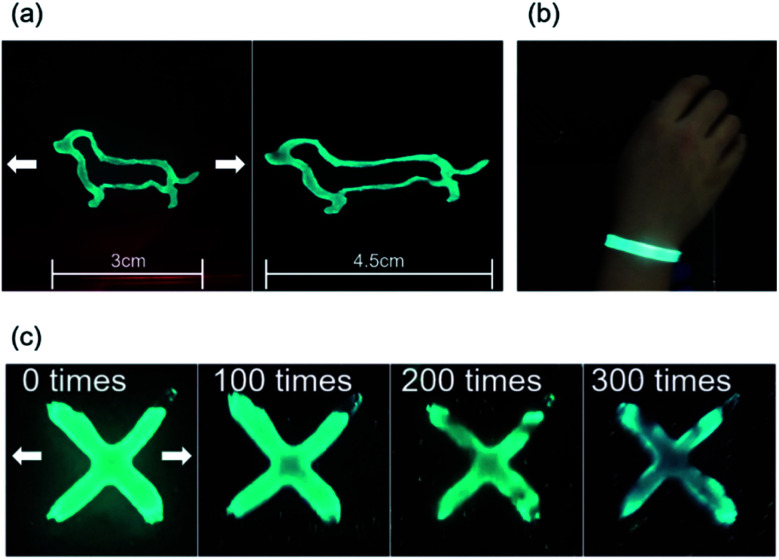
(a) Photography of brush painted EL device before and after stretching, (b) EL device wrapped around human wrist. (c) Stretching fatigue test with EL device.

Electroluminescence (EL) devices were fabricated from the Ag NW/PEDOT:PSS electrode as shown in Fig. 6. When an AC voltage was introduced into the EL device composed of AP electrode and phosphor layer, electrons of phosphor layer get excited by the intensity of the electric field generating in the fluorescent layer. When the excited electrons returned to a stable state, the device emitted light. Fig. 6a shows the schematic image of ZnS:Cu and PDMS mixed in a weight ratio of 2 : 1 respectively as a brush paintable phosphor ink. The fabrication and the number of layers of the EL devices are illustrated in Fig. 6b and c. The PDMS layer in the electrode worked as an insulating layer which protected the device from short circuit. Fig. 7a shows the phosphor ink brush painted on the AP electrode to show the canine image in both normal and stretched positions. We also made a wearable EL device which can be used as a safety vest or strap for a night worker or riders as shown in Fig. 7b. The EL devices were stretched at 20% for 300 times without losing their luminescence property, as shown in Fig. 7c. Despite the fact that the brightness decreased slightly as the number of repetitions increased, the EL device was still operational and easily distinguishable from the non-phosphor area.

**Fig. 8:**
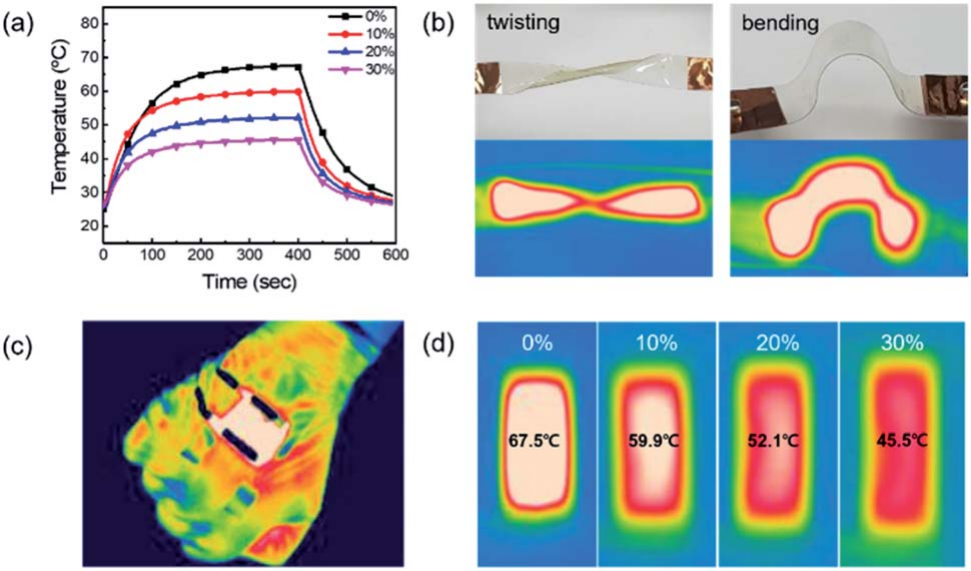
(a) Temperature profile of the stretchable thin film heater as a function of strain rate. (b) Thin film heater on twisted and curved state. (c) IR image of wearable thin film heater attached to hand and (d) according to the strain rate at applied voltage of 7 V.

Fig. 8a shows the temperature profile of a TFH made of AP electrode with respect to strain rate. The TFH device attained 67.5 °C without strain and 45.5 °C with 30% stretch. This temperature change can be expressed by following equation:^47^

4 *V*^2^/*RA*(*T*_s_ − *T*_i_) = *h*_c_

In eqn (4), *V* is voltage, *h*_c_ is convective heat transfer constant and *T*_i_ is the initial temperature of TFHs. They are constant with increasing strain rate. *R* is resistance of electrode and *A* is area of TFH. The resistance increased when the strain rate increased, and this increased value made the saturation temperature *T*_s_ decrease. However, the performance of stretched TFH was still enough to function as a heater. In Fig. 8b, the photographs show twisted and bent TFH and IR images at twisted and bent states. These images demonstrated that, even after mechanical stress was applied to the electrode, heat was uniformly distributed throughout the heater. This was due to the high flexibility of AP electrode. Fig. 8c shows the IR image of wearable TFH and illustrates that AP electrode is a good candidate for wearable TFH. During stretching, a voltage of 7 V was applied to TFH. At 0% strain rate, the saturation temperature was 67.5 °C whereas at 30% strain rate the saturation temperature was 45.5 °C. During TFH stretching, the area of the TFH device increased, resulting in a decrease in the concentration of Ag NW and PEDOT:PSS per area. From eqn (4), the temperature decreased with increasing strain rate. However, overall heating during the stretching did not do any harm.

## 4. Conclusions

In this study, we demonstrated that mixing PEDOT:PSS into Ag NW enhanced the stretchability of Ag NW electrode by conductive bridge effect where the added PEDOT:PSS interlinked with the disconnected Ag NW network. The electrode was fabricated using bar coater with optimized AP ink on PU substrate and spin coater with PDMS/*n*-hexane mixture on the electrode surface. The optical, electrical, and mechanical properties of an electrode were investigated. The optimized AP electrode has 14.93 Ω sq^−1^. of sheet resistance and 88.6% of transmittance at 550 nm wavelength. The PDMS protective layer has a low surface energy and a hydrophobic property, which can protect the electrode from humidity-induced washing and corrosion. In the dynamic stretching test, the AP electrode endured over 40% stretch strain rate and 20% stretching repetition test for more than 350 times. These results showed enhanced endurance for stretching when compared with bare Ag NW electrodes. Other mechanical tests such as bending, folding, twisting, and rolling showed remarkable performances. For applications of electrode, the AP electrode was fabricated as EL devices and TFH devices. Various designed EL devices were created using brush paintable PDMS/ZnS:Cu phosphor ink. After the stretching cycle test, these devices performed well when stretched and worn 300 times. Moreover, TFH performed effectively in the twisted and bending states. These results demonstrated the potential of AP electrodes in a variety of stretchable or wearable applications.

## Conflicts of interest

There are no conflicts to declare.

## Supplementary Material
